# Immucillins Impair *Leishmania (L*.*) infantum chagasi* and *Leishmania (L*.*) amazonensis* Multiplication *In Vitro*

**DOI:** 10.1371/journal.pone.0124183

**Published:** 2015-04-24

**Authors:** Elisangela Oliveira Freitas, Dirlei Nico, Rong Guan, José Roberto Meyer-Fernandes, Keith Clinch, Gary B. Evans, Peter C. Tyler, Vern L. Schramm, Clarisa B. Palatnik-de-Sousa

**Affiliations:** 1 Departamento de Microbiologia Geral, Instituto de Microbiologia Paulo de Góes, Universidade Federal do Rio de Janeiro, Rio de Janeiro, Brazil; 2 Department of Biochemistry, Albert Einstein College of Medicine, Yeshiva University, New York, United States of America; 3 Instituto de Bioquímica Médica, Universidade Federal do Rio de Janeiro, Rio de Janeiro, Brazil; 4 The Ferrier Research Institute, Victoria University of Wellington, Wellington, New Zealand; National Center for Cell Science, INDIA

## Abstract

Chemotherapy against visceral leishmaniasis is associated with high toxicity and drug resistance. *Leishmania* parasites are purine auxotrophs that obtain their purines from exogenous sources. Nucleoside hydrolases release purines from nucleosides and are drug targets for anti-leishmanial drugs, absent in mammal cells. We investigated the substrate specificity of the *Leishmania (L*.*) donovani* recombinant nucleoside hydrolase NH36 and the inhibitory effect of the immucillins IA (ImmA), DIA (DADMe-ImmA), DIH (DADMe-ImmH), SMIH (SerMe-ImmH), IH (ImmH), DIG (DADMe-ImmG), SMIG (SerMe-ImmG) and SMIA (SerME-ImmA) on its enzymatic activity. The inhibitory effects of immucillins on the *in vitro* multiplication of *L*. *(L*.*) infantum chagasi* and *L*. *(L*.*) amazonensis* promastigotes were determined using 0.05–500 μM and, when needed, 0.01–50 nM of each drug. The inhibition on multiplication of *L*. *(L*.*) infantum chagasi* intracellular amastigotes *in vitro* was assayed using 0.5, 1, 5 and 10 μM of IA, IH and SMIH. The NH36 shows specificity for inosine, guanosine, adenosine, uridine and cytidine with preference for adenosine and inosine. IA, IH, DIH, DIG, SMIH and SMIG immucillins inhibited *L*. *(L*.*) infantum chagasi* and *L*. *(L*.*) amazonensis* promastigote growth *in vitro* at nanomolar to micromolar concentrations. Promastigote replication was also inhibited in a chemically defined medium without a nucleoside source. Addition of adenosine decreases the immucillin toxicity. IA and IH inhibited the NH36 enzymatic activity (*Ki* = 0.080 μM for IA and 0.019 μM for IH). IA, IH and SMIH at 10 μM concentration, reduced the *in vitro* amastigote replication inside mice macrophages by 95% with no apparent effect on macrophage viability. Transmission electron microscopy revealed global alterations and swelling of *L*. *(L*.*) infantum chagasi* promastigotes after treatment with IA and IH while SMIH treatment determined intense cytoplasm vacuolization, enlarged vesicles and altered kinetoplasts. Our results suggest that IA, IH and SMIH may provide new chemotherapy agents for leishmaniasis.

## Introduction

Visceral leishmaniasis (VL) is a chronic and often lethal human and canine vector-borne disease caused by protozoa parasites of the *Leishmania* genus. In Brazil, the infection is caused by *L*. *(L*.*) infantum chagasi*. Human VL is the most severe form of the disease and is lethal if not treated early after the onset of clinic and pathological signs [1]. Human cutaneous leishmaniasis (CL) is a more frequent but less severe form of leishmaniasis [2]. In Brazil, CL is caused mainly by *L*. *(V*.*) braziliensis* and *L*. *(L*.*) amazonensis* [3]. Infection by *L*. *(L*.*) amazonensis* shows varied clinical forms including localized cutaneous and anergic diffuse cutaneous leishmaniasis, but *L*. *(V*.*) braziliensis* infection can cause localized, disseminated and also disfiguring mucocutaneous leishmaniasis [3]. Both VL and CL are expanding to new areas of the globe due to changes in the insect vector habitats. They are also opportunistic diseases in individuals suffering from AIDS [4]. The life cycle of *Leishmania* parasites includes promastigote forms, which inhabit the sand fly and are transmitted to humans, dogs and rodents. After sand fly transmission, amastigotes replicate inside macrophages [4].

The control of VL has been accomplished by treatment of human cases, sacrifice of infected dogs, and insecticide treatment of residences [1, 5]. Few drugs are available for treatment of leishmaniasis: pentavalent antimonium (meglumine antimoniate and sodium stibogluconate), pentamidine, amphotericin B, liposomal amphotericin B, miltefosine and paromomoycin [6–9]. Most treatments require hospitalization, show high toxicity [6–8] and an overall case-fatality rate of 10% [6]. In India, where 70% of the cases of VL occur, a 65% of failure rate is reported for antimonial treatments [10]. Current drug therapy of dogs attempts to achieve reservoir for human infection, and is performed with the same drugs used for human therapy. Its effects are controversial and it increases the probabilities of selection of resistant parasite strains [11]. The search of alternative drugs of high therapeutic efficacy and low toxicity is mandatory.

In order to establish a successful infection, *Leishmania* and other protozoa parasites have developed efficient mechanisms for rapid synthesis of DNA and replication. Protozoa parasites including *Leishmania* [12, 13], *Plasmodium* [14, 15], *Entamoeba histolytica* [16, 17], *Giardia lamblia* [18], *Toxoplasma gondii* [19, 20] *Trypanosoma* [21, 22] and a few bacteria and fungi [23] are purine auxotrophs, that obtain their purines from exogenous precursors through purine salvage pathways [24]. In cells, nucleosides are hydrolyzed by nucleoside hydrolases (NH) [13] or undergo phosphorolysis by purine nucleoside phosphorylase (PNP) which release the purine bases to be used in parasite DNA synthesis [24]. Nucleoside hydrolases are absent in human cells, and are therefore potential targets of differential toxicity. Inhibitors of NHs would impede the parasite purine salvage with less effect on the human or animal host’s cell, where *de novo* purine synthesis prevails [25, 26].

Immucillin A and Immucillin H are synthetic deazapurine iminoribitols first described in the 90´s as nanomolar inhibitors of the *in vitro* activity of the nucleoside hydrolases of *Leishmania (L*.*) major* [13], *Crithidia fasciculata* [27] and *Trypanosoma brucei brucei* [21], but were never reported to be antiparasitic. More recently, second and third generation immucillins have been developed as inhibitors of PNP [28] and induce purine–starvation and death of *Plasmodium falciparum* in monkeys suffering from malaria [14].

Considering the urgency in identifying new chemical compounds with potential cure activity on all forms of leishmaniasis, we screened the inhibitory effect of the immucillins: IA (ImmA), DIA (DADMe-ImmA), DIH (DADMe-ImmH), SMIH (SerMe-ImmH), IH (ImmH), DIG (DADMe-ImmG), SMIG (SerMe-ImmG) and SMIA (SerMe-ImmA) on the *in vitro* multiplication of *Leishmania (L*.*) infantum chagasi* and *Leishmania (L*.*) amazonensis* promastigotes. We also assayed their inhibitory effect on the *in vitro* activity of the recombinant *Leishmania donovani* nucleoside hydrolase (NH36) [29–31] and defined its substrate specificity. We identified three potent immucillins that efficiently interrupt the multiplication of promastigote and intracellular amastigote *in vitro*. Two of them inhibit the cleavage of nucleosides by the nucleoside hydrolase, at nanomolar concentrations in the absence of toxicity to the murine macrophage host cell. A third inhibits parasite growth, but is not a powerful NH inhibitor, suggesting a distinct mechanism.

## Methods

### Parasites

The strain of *Leishmania (L*.*) infantum chagasi* MHOM/BR/1974/PP75 was obtained from the *Leishmania* Type Culture Collection (Fundacão Oswaldo Cruz, Rio de Janeiro, RJ, Brazil) and the strain of *Leishmania (L*.*) amazonensis* (MHOM/BR/1975/Josefa) was kindly given by Professor Rosângela de Araújo Soares from Universidade Federal do Rio de Janeiro. Promastigotes were maintained by weekly transfers in screw-capped glass tubes containing Schneider’s insect medium (Sigma Aldrich, MO, USA), pH 7.0, supplemented with 10% Fetal Bovine Serum (FBS) at 26°C.

### Enzyme Expression and Purification

BL21DE3 *E*.*coli* were transformed with the synthetic plasmid pET 28b encoding the gene of NH36 of *Leishmania (L*.*) donovani* (EMBL, Genbank and DDJB data bases, access number AY007193) [30] with a His_6_tag at the C-terminal [31]. Expression was induced with 0.5 mM IPTG and overnight incubation at 20°C. Purification of NH36 was performed on a column of Ni-NTA Superflow resin (Qiagen, USA) eluted by a stepwise imidazole gradient and monitored by SDS-polyacrylamide gel electrophoresis and protein assay. The extinction coefficient of NH36 is 21.437 mM^-1^ cm^-1^ at 280 nm, as calculated with the ProtParam program from ExPASy, and was used to estimate protein concentration (http://ca.expasy.org/seqanalref/). The yield of NH36 after purification was 11.87 mg per Liter of bacterial growth culture. Non-specific His-proteins with weak interactions were eluted with 50–100 mM imidazole (S1 Fig). The purified NH36 started to elute with 150 mM imidazole. We used 250 mM of imidazol in elution buffer for purification of NH36.

### Substrate Activity Assay

Steady-state kinetic rates for NH36 were determined on the substrates inosine, guanosine, adenosine, uridine and cytidine. Hypoxanthine and adenine formation were coupled to xanthine oxidase (12.9 mM^-1^ cm^-1^ at 293 nm and 15.2 mM^-1^ cm^-1^ at 305 nm, respectively), and the others were followed by direct uv absorbance changes resulting from cleavage of the N-ribosidic bond. Reaction mixtures contained 50 mM Hepes (pH 7.4), 100 mM NaCl and 5 mM of DTT. One unit, and 0.1 unit of xanthine oxidase (XOD) were used as the coupling enzyme to the adenosine and inosine assays, respectively. Spectral changes for hydrolysis of guanosine, uridine and cytidine were 5.4 mM^-1^ cm^-1^ at 257 nm,1.8 mM^-1^ cm^-1^ at 280 nm and 1.8 mM^-1^ cm^-1^ at 288 nm, respectively. Variable concentrations of substrate and appropriate amounts of purified NH36 were added in all assays. The hydrolysis of nucleosides to bases and ribose was followed by continuous recording of optical absorbance after addition of the enzyme, in a CARY 300 UV-visible spectrophotometer. Control rates with no enzyme addition were subtracted from initial rates. Reactions with substrates were conducted at 25°C using 1 cm path length cuvettes containing 1 mL reaction volumes. Kinetic parameters of NH36 were obtained by fitting initial rates to the Michaelis−Menten equation using GraFit 5 (Erithacus Software).

### *K*_i_ determination in the presence of immucillins

**Fig 1:**
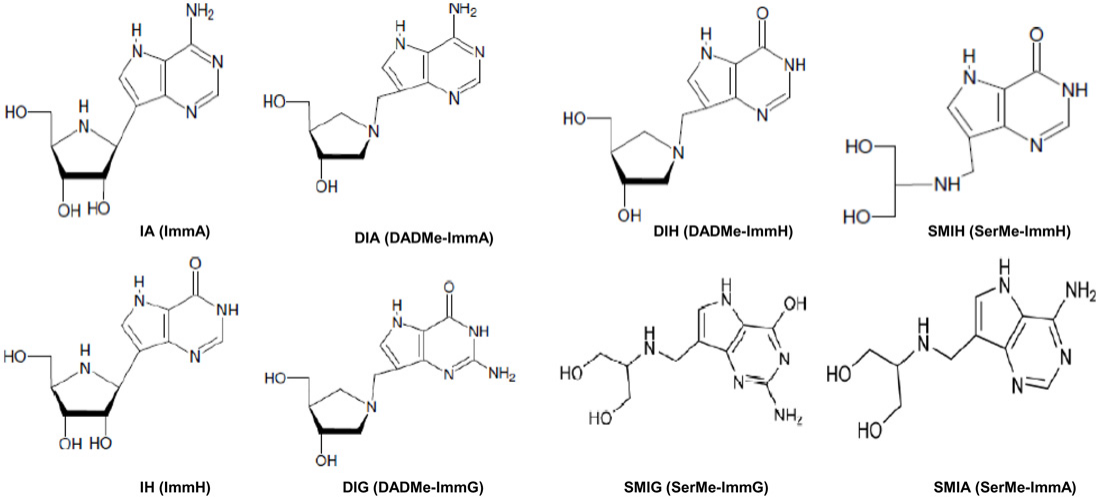
Chemical structure of the immucillins assayed for inhibition of *Leishmania* multiplication: IA (ImmA), DIA (DADMe-ImmA), DIH (DADMe-ImmH), SMIH (SerMe-ImmH), IH (ImmH), DIG (DADMe-ImmG), SMIG (SerMe-ImmG) and SMIA (SerMe-ImmA).

The inhibition of NH36 activity by each one of the eight immucillins (Fig 1) was conducted by adding 0.5 nM NH36 to reaction mixtures at room temperature, in 1 cm path length cuvettes in a total reaction volume of 1mL. The reaction mixtures contained 10 mM inosine as substrate, 50 mM Hepes (pH 7.4), 100 mM KCl, 5 mM DTT and 1 unit XOD. The hydrolysis of inosine to hypoxanthine and ribose was followed by continuous reading of optical absorbance at 293 nm, using variable concentrations of inhibitors in a CARY 300UV-visible spectrophotometer. Controls without enzyme and without inhibitors were included in all inhibition assays. The inhibition constant for each immucillin was obtained, using GraFit 5 (Erithacus Software), by fitting initial rates with variable inhibitor concentrations to the equation (v_i_/v_0_ = [S] / K_m_ + [S] + K_m_[I] / K_i_),, where *v*_i_ is the initial rate in the presence of the inhibitor, *v*_0_ is the initial rate in the absence of the inhibitor, *K*_m_ is the Michaelis constant for inosine, [S] and [I] are inosine and inhibitor concentrations, respectively, and *Ki* is the inhibition constant.

### Inhibition on the *in vitro* growth of *Leishmania* promastigotes by immucillins

Stock solutions (20 mM) of immucillins IA (ImmA), DIA (DADMe-ImmA), DIH (DADMe-ImmH), SMIH (SerMe-ImmH), IH (ImmH), DIG (DADMe-ImmG) SerMe-ImmG and SerMe-ImmA (Fig 1) were prepared using Milli-Q purified water. Log phase promastigotes of *L*. *(L*.*) infantum chagasi* and *L*. *(L*.*) amazonensis* (5 × 10^4^ viable promastigotes/mL) were incubated with 0.05, 0.1, 1.0, 5.0, 10, 25, 50, 250 and 500 μM of each immucillin and, when needed, also with 0.01, 0.1, 0.5, 1, 5, 10, 25 and 50 nM in FBS supplemented Schneider’s insect medium. Promastigotes were recorded in a Neubauer chamber after 24, 48, 72 and 96 hours of incubation at 26°C. The 50% inhibitory concentration (IC50) was evaluated after 48 hours [32, 33] by linear interpolation, using the Excel computer software and the formula IC50 = C_A_ + [(B-C_A_) x ((N control/2)-N_A_))/ (N_A_-N_B_)], where C is concentration, N is the number of promastigotes and A and B are the points between which 50% of inhibition occurs. Alternatively, we assessed the inhibitory effect of IA, IH and SMIH on *L*. *(L*.*) infantum chagasi* growth in a daily addition protocol (0, 24 and 48 h), and of IA using the chemically defined RPMI liquid medium (Sigma Co.) which lacks nucleotides, nucleosides or nucleobases. We also assayed the possibility of reversing the inhibitory effect of IA in RPMI medium by the addition of adenosine to the culture medium. The concentration of adenosine in Schneider´s insect serum supplemented medium was assessed by HPLC using a C-18 reverse-phase column as described [34].

### Electron microscopy

Promastigotes of *L*. *(L*.*) infantum chagasi* were incubated in the absence or presence of the immucillins IA, IH and SMIH at a 10 μM concentration, for 72 h, fixed in 2.5% glutaraldehyde, formaldehyde 4% in 0.1 M cacodylate buffer, pH 7.2, washed with cacodylate buffer, and post fixed for 1 h in 0.1 M cacodylate buffer containing 1% osmium tetroxide and 1.25% potassium ferrocyanide. The cells were washed with Milli Q water, dehydrated in increasing concentration of acetone and embedded in Epon (1: 1) (PolyBed 812). The ultrathin sections were obtained, contrasted with 5% uranyl acetate and lead citrate and observed under a Joel 1200 electron microscope.

### Macrophages viability and chemotherapeutic effects of immucillins on the intracellular amastigote forms of *L*. *(L*.*) infantum chagasi*

Peritoneal macrophages [35] were obtained from BALB/c mice females and treated with IA, IH or SMIH or Glucantime at a 10 μM concentration or with no additions, washed after 48 h incubation and their viability assessed with 3% Trypan Blue [35]. Alternatively, the immucillins were added at 0, 24 and 48 h and viability assessed after 72h. The concentration that caused 50% of macrophage cytotoxicity (CC50) was determined by linear interpolation, after addition of the drugs (10 μM—5 mM) at 0h and incubation for 48 h. For chemotherapy assays, peritoneal macrophages [36] were incubated overnight, infected with promastigotes of *L*. *(L*.*) infantum chagasi* during 2 h, washed, incubated for additional 24 h and treated with a single 10 μM dose of IA, IH and SMIH (0 h), or with daily doses added at 0, 24 and 48h. The amastigote growth was assessed 24 h after the last drug addition, on 200 cells in Giemsa stained coverslips and expressed as phagocytic index = percent of infected macrophages *x* average number of intracellular amastigotes. The effective concentration of drugs at which intracellular amastigote replication is inhibited by 50 percent (EC50) was determined by linear interpolation, after addition of the drugs (0.5–10 μM) at 0h and incubation for 24 h. All experiments with mice were reviewed and approved by the Animal Care and Use Committee of the Instituto de Biofísica Carlos Chagas Fo.-UFRJ (CAUAP-CONCEA, Brazil, IMPPG-016) and were performed according to the guidelines of the National Institutes of Health, USA. We made all efforts in order to minimize animal suffering.

### Statistical analysis

For comparison of means we used the non-parametrical Kruskall Wallis and Mann Whitney tests and IC95% (GraphPad Prism6 program)

## Results

### Substrate Specificity

The nucleoside hydrolase of *L*. *(L*.*) donovani* demonstrates the characteristics expected for nonspecific nucleoside hydrolases (Table 1), including the recognition of both adenosine and inosine as favored substrates and good catalytic activity with the pyrimidine nucleosides uridine and cytidine. Comparing the kcat/Km values (the catalytic efficiency), guanosine, on the other hand, is not a good substrate. NH36 of *Leishmania (L*.*) donovani* is therefore a nonspecific nucleoside hydrolase.

**Table 1:** Kinetic parameters for Nucleoside hydrolase NH36 of *L*. *(L*.*) donovani*.

Substrate	*K*_*m*_ (μM)	*k*_*cat*_ s ^-1^	*k*_*cat*_ */K*_*m*_ (M^-1^s^-1^)
**Inosine**	340 ± 30	10.8 ± 0.3	31.8 x 10^3^
**Guanosine**	380 ± 153	0.47 ± 0.1	1.2 x 10^3^
**Adenosine**	70 ± 30	3.7 ± 0.1	5.3 x 10^4^
**Uridine**	2,100 ± 561	34.3 ± 4.2	1.6 x 10^4^
**Cytidine**	1,403 ± 282	13.1 ± 1.1	9.3 x 10^3^

Initial reaction rates were measured under conditions described under “Materials and Methods.” The kinetic parameters and associated errors were determined with fits of the data to the Michaelis-Menten equation.

### Effect of immucillins on the growth of promastigotes of *Leishmania* and on nucleoside hydrolase activity

**Fig 2:**
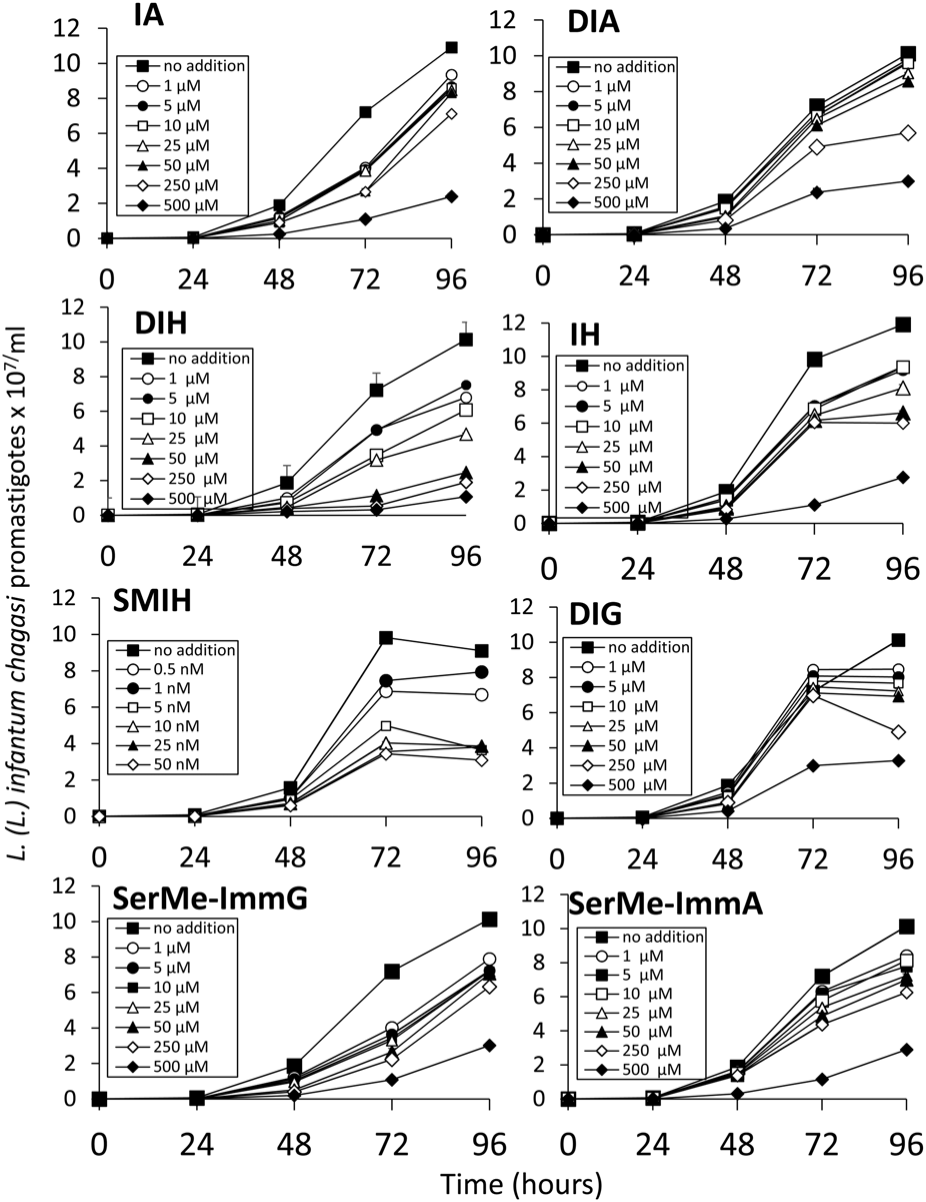
Effect of immucillins IA, DIA, IH, DIH, IH, SMIH, DIG, SerMe-ImmG and Ser-Me ImmA on the growth rate of *Leishmania (L.) infantum chagasi* in acellular medium. The growth of parasites cultured at 26°C in the absence (no addition control) or the presence of the immucillins assayed in concentrations ranging from 1 to 500 μM and in the presence of immucillin SMIH tested in concentrations from 0.05 to 50 nM. The inhibitors were added at 0 hour and the parasites were counted daily. The *y* axis represent the number of promastigotes x 10^7^/ml of liquid medium. Data shown are the mean ± SE of two independent experiments performed in triplicate.

**Fig 3:**
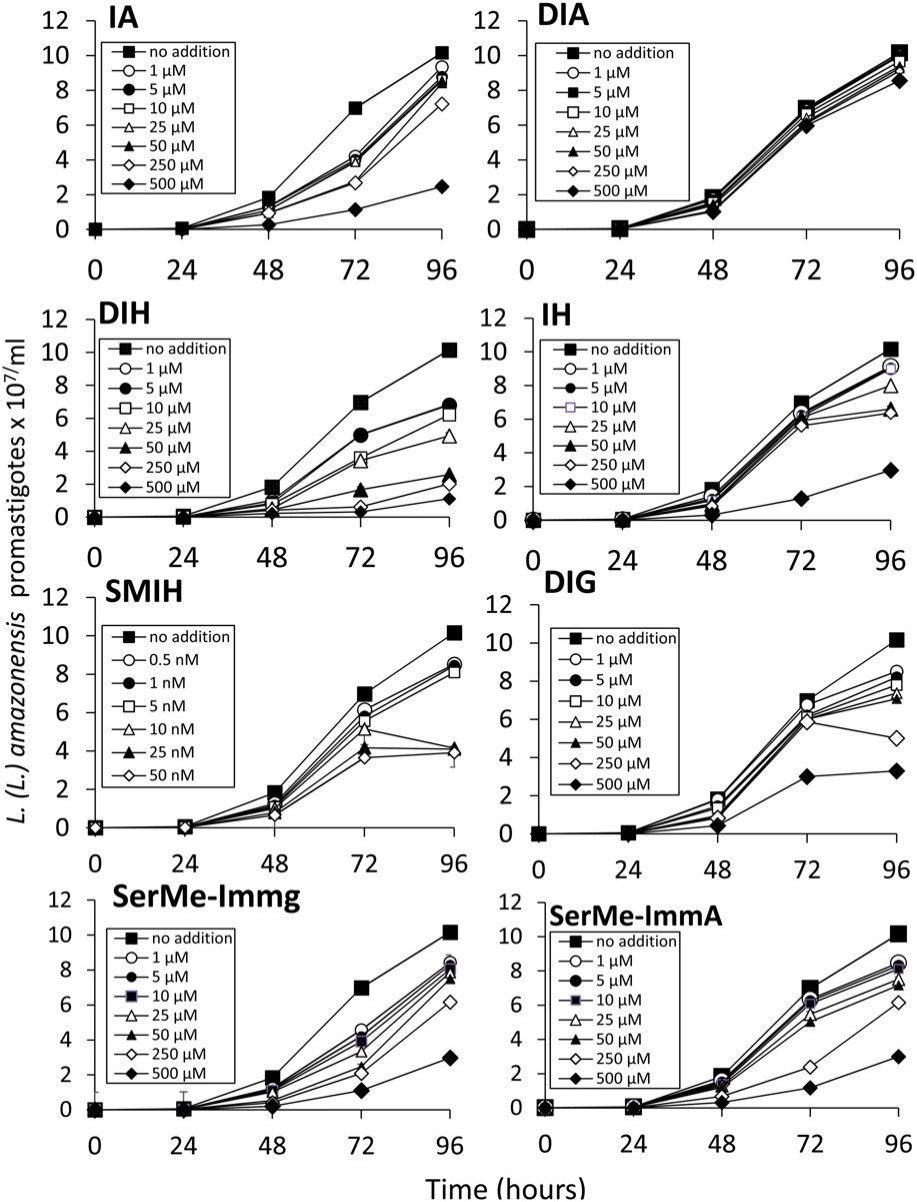
Effect of immucillins IA, DIA, IH, DIH, IH, SMIH, DIG, SerMe-ImmG and Ser-Me ImmA on the growth rate of *Leishmania (L.) amazonensis* in acellular medium. The growth of parasites cultured at 26°C in the absence (no addition control) or the presence of the immucillins assayed in concentrations ranging from 1 to 500 μM and in the presence of immucillin SMIH tested in concentrations from 0.05 to 50 nM. The inhibitors were added at 0 hour and the parasites were counted daily. The *y* axis represent the number of promastigotes x 10^7^/ml of liquid medium. Data shown are the mean ± SE of two independent experiments performed in triplicate.

All parasite growth inhibitions were determined in a time- and dose-dependent manner (Figs 2 and 3). Similar potency was detected for the immucillins SMIH, IA and SerMe-ImmG, on the multiplication of both *L*. *(L*.*) infantum chagasi* and *L*. *(L*.*) amazonensis* (Figs 2 and 3 and Table 2). DIH, IH and DIG, on the other hand, although active against both species, were more potent against *L*. *(L*.*) infantum chagasi*, while SMIA induced stronger inhibition on *L*. *(L*.*) amazonensis*. Furthermore, DIA was active against *L*. *(L*.*) infantum chagasi* (Fig 2 and Table 2) but not against *L*. *(L*.*) amazonensis*. Only SMIH reached its IC50 on *L*. *(L*.*) infantum chagasi* and *L*. *(L*.*) amazonesis* promastigotes, at nanomolar concentration (Table 2) while DIH, IA, IH, DIG and SerMe-ImmG needed concentrations ranging from 1.8–49.7 μM. DIA and SerMe-ImmA were less active on growth inhibition (Figs 2 and 3 and Table 2). After 96 h of culture with 500 μM of immucilin the percent inhibition on *L*. *(L*.*) infantum chagasi* promastigote growth was 78% for IA, 69% for DIA, 89% for DIH, 99% for IH, 68% for DIG, 70% for SerMe-ImmG and 71% for SerMe-ImmA. SMIH induced 99% of inhibition at 50 nM concentration (Fig 2). Also at 500 μM, the percent inhibition on *L*. *(L*.*) amazonensis* replication was 76% for IA, 16% for DIA, 89% for DIH, 71% for IH, 67% for DIG and 71% for Serme-ImmH and for SerMe-ImmA while SMIH induced a 61% of growth inhibition at 50 nM.

**Table 2:** Immucillin effects on the multiplication of *Leishmania* promastigotes *in vitro* and on the activity of Nucleoside hydrolase NH36.

Immucillin	IC50 (CI 95%) on *L*. *(L*.*) infantum chagasi* promastigotes *in vitro* growth (μM)	IC50 (CI 95%) on *L*. *(L*.*) amazonensis* promastigotes *in vitro* growth (μM)	*Ki* (μM)
**SMIH (SerMe-ImmH)**	0.008 (0.007–0.009)	0.007 (0.005–0.008)	> 5
**DIH (DADMe-ImmH)**	1.8 (0.9–2.7)	3.4 (2.2–4.6)	> 5
**IA (ImmA)**	23.9 (-3.7–51.4)	48.7 (43.1–54.4)	0.080
**DIA (DADMe-ImmA)**	109.0 (57.8–157.8)	-	> 5
**IH (ImmH)**	30.6 (20.7–40.6)	49.7 (34.8–64.6)	0.019
**DIG (DADMe-ImmG)**	23.7 (23.1–24.4)	40.0 (29.8–50.2)	> 5
**SMIA (SerMe-ImmA)**	349.1 (328.1–370.1)	156.8 (120.0–193.5)	>5
**SMIG (SerMe-ImmG)**	25.8 (21.7–30.0)	29.45 (25.5–33.4)	1.80

The best inhibitors of the nucleoside hydrolase activity *in vitro* were IA and IH (Table 2) which, inhibited both, the parasite multiplication and the enzyme activity (*K*i = 0.080 μM and 0.019 μM, respectively). In spite of the low IC50 values for SMIH, DIH, DIG and SerMe-ImmG on both *Leishmania* species, their relatively high *K*i values (1.80 or >5 μM) indicate the inhibition of growth is not related to inhibition of NH36 enzymatic activity. Finally, the DIA (DADMe-ImmA) and SerMe-ImmA, which did not impair the parasite growth at low concentrations, also did not inhibit the NH36 function (*K*i >5 μM) (Table 2).

**Fig 4:**
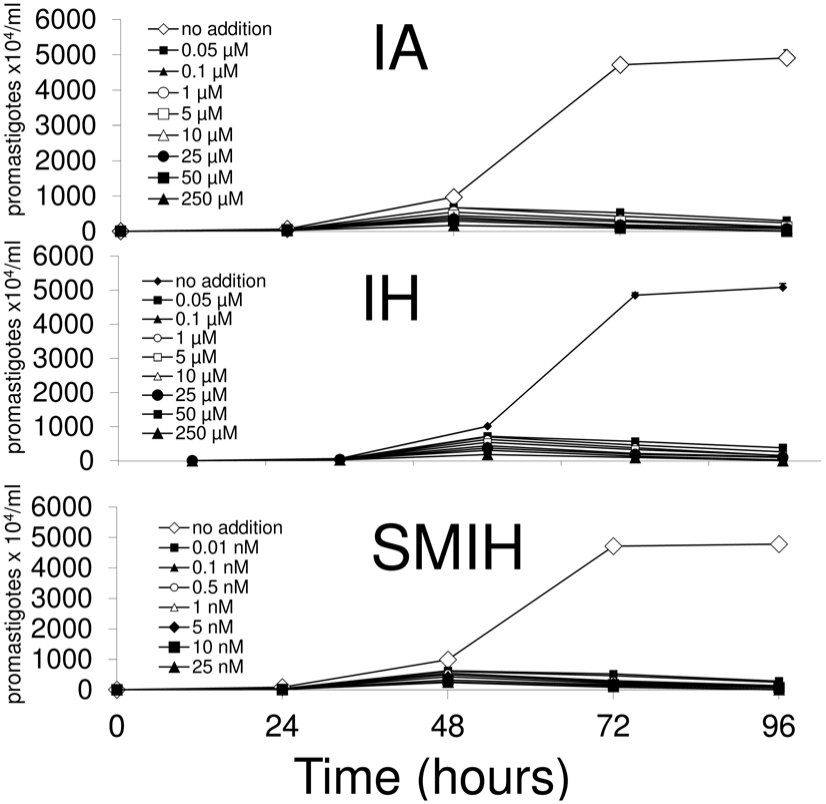
Effect of daily addition of immucillins on *L*. *(L*.*) infantum chagasi* promastigote replication. Immucillins IA, IH (0.05 to 250 μM concentration) and SMIH (0.01nM to 25nM concentration) were added in culture at 0h, 24h and 48h. The parasite replication was monitored daily. Promastigotes with no addition were included as control. The *y* axis represents the number of promastigotes x 10^4^/ml. Data shown are the mean ± SE of two independent experiments performed in triplicate.

We additionally assayed the inhibitory potential of IA, IH (most potent inhibitors of NH36 activity) and SMIH (most potent inhibitor of promastigote replication) on *L*. *(L*.*) infantum chagasi* promastigote growth after the successive addition of the drugs at 0, 24 and 48 h (Fig 4). Using this protocol, we observed a significantly high reduction of promastigote growth after 48h. When compared to the effect of a single addition (Table 2), the daily addition reduced the IC50 by 91% after incubation with IA (IC50 = 3.6 μM), by 83% after incubation with IH (IC50 = 6.7 μM) and by 88% after incubation with SMIH (IC50 = 0.001 μM). Strongest inhibitions were detected at 72 and 96 h, when the IC50 values were lower than 0.05 μM for IA and IH and lower than 0.01 nM for SMIH (Fig 4A, 4B and 4C respectively).

**Fig 5:**
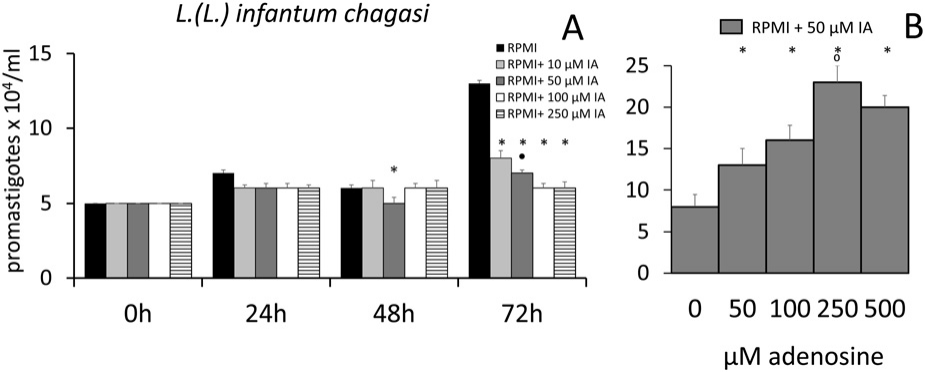
Effect of immucillins IA on multiplication of promastigotes of *L*. *(L*.*) infantum chagasi* in a chemically defined culture medium. (A) Parasites were cultured at 26°C in RPMI medium with no addition, or with 10, 50, 100 and 250 μM immucillin IA and counted daily. (B) Parasites were cultured at 26°C in RPMI medium with 50 μM IA and with 50, 100, 250 or 500 μM adenosine, or with no addition, and counted after 72h of incubation. The *y* axis represent the number of promastigotes x 10^4^/ml of liquid medium. Data shown are the mean ± SE of two independent experiments performed in triplicate. * significant differences from the RPMI controls (p < 0.005), ● significant difference from the RPMI + 50 μM of immucillin IA (p < 0.005). ● significant difference from all groups treated with adenosine and 50 μM of immucillin IA.

As a control, we also studied the multiplication of *L*. *(L*.*) infantum chagasi* in the chemically defined RPMI medium, which lacks nucleosides or nucleobases (Fig 5). Promastigotes developed a normal growth curve and the inhibitory effect of 50 μM IA was already evident at 48 h of culture. At 72 h, all concentrations of IA were inhibitory. When compared to RPMI with no addition, incubation with 10 μM and 50 μM of IA induced 38% and 46% of inhibition, respectively, while the IC50 was 75 μM (Fig 5A). These results suggest that in conditions without exogenous purine supply, IA inhibits parasite replication. Additionally, the inhibition of parasite growth by IA can be reversed by the presence of exogenous adenosine in the culture medium (Fig 5B). Promastigote replication in RPMI medium was less intense than in the Schneider´s supplemented insect medium (Fig 2) likely related to the presence of nucleosides in the FBS supplemented Schneider´s medium. Supporting this hypothesis, using HPLC analysis, we were able to identify, the presence of 0.4 μM adenosine in Schneider’s medium (S2 Fig).

### Effect of immucillins on the ultrastructure of promastigote forms of *L*. *(L*.*) infantum chagasi*

**Fig 6:**
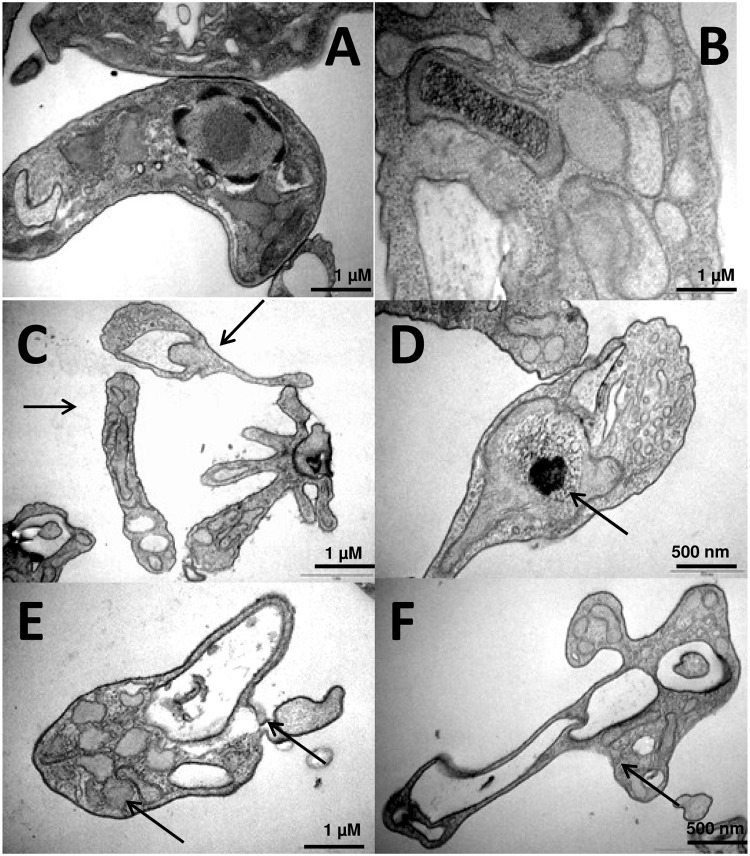
Effect of treatment with immucillins IA, IH and SMIH on the cellular ultrastructure of *L*. *(L*.*) infantum chagasi* promastigotes. Promastigotes were cultured in the absence (A, B) or in the presence of the immucillins IA (C), IH (D) or SMIH (E and F) at a 10 μM concentration and examined by transmission electron microscopy. The untreated controls show the intact full cell structure. Cells treated with the inhibitors show morphological alterations.

We also assessed the impact effect of IA, IH and SMIH, at 10 μM concentration, on the ultrastructure of *L*. *(L*.*) infantum chagasi* promastigotes, by transmission electronic microscopy. Fig 6 illustrates the morphology of promastigotes after 72 h of culture with the inhibitors. While the untreated controls (Fig 6A and 6B) show a normal cellular ultrastructure, promastigotes treated with IA (Fig 6C, arrows) show global alterations on their cellular morphology. Treatment with IH (Fig 6D arrows) caused alterations and swelling of kinetoplasts, while promastigotes treated with SMIH (Fig 6E, arrows) showed complete alteration of their cellular morphology with intense cytoplasm vacuolization, enlarged vesicles and alterations in the kinetoplasts (Fig 6F, arrows).

### Macrophages cytotoxicity and chemotherapeutic effects of immucillins on amastigote forms of *L*. *(L*.*) infantum chagasi*.

**Fig 7:**
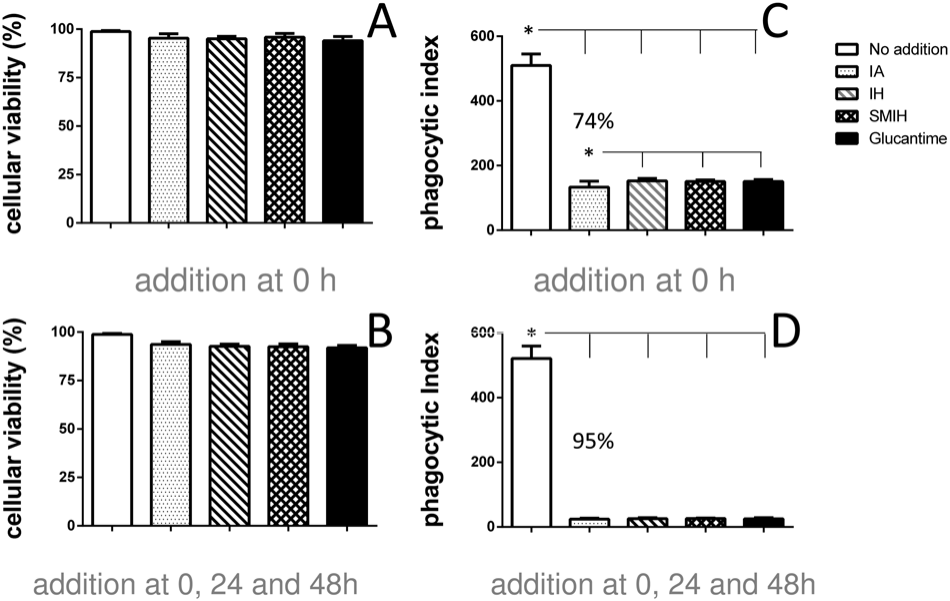
Effect of immucillins IA, IH and SMIH on the replication of intracellular amastigotes of *L*. *(L*.*) infantum chagasi*. Immucillins and Glucantime were added at 0 hour (A), at a 10μM concentration, to peritoneal macrophages incubated *in vitro* and macrophage viability was assessed by the method of Trypan blue at 48h, or the drugs were added at 0, 24 and 48 h (B) and viability was assessed at 72h. Peritoneal macrophages were infected with promastigotes of *L*. *(L*.*) infantum chagasi* and a dose of each immucillin (10 μM) was added at 0 hour (C) or at 0, 24 and 48 h (D) and the phagocytic index (percent of infected macrophages *x* average number of intracellular amastigotes) was determined at 72h. Data shown are the mean ± SE of two independent experiments performed in triplicate. Macrophages treated with 10 μM Glucantime were used as controls.

We assessed the cellular viability of BALB/c intraperitoneal macrophages after *in vitro* culture with IA, IH or SMIH at a 10 μM concentration. We used 10 μM of Glucantime as control. We show the percent of viable macrophages, after *in vitro* culture for 48 h, with immucillins added at 0 h (Fig 7A) or after incubation for 72 h, with immucillins added daily, at 0, 24 and 48 h (Fig 7B). None of the treatments damaged macrophage viability and no significant difference was detected between treated and untreated macrophages, which showed a 100% of viability. The CC50 determination confirmed the low citotoxicity of the drugs (Table 3) which were lethal to 50% macrophages only at concentrations ranging from 800–950 μM. The means of IA, IH and SMIH fall outside the IC95% of Glucantime IA indicating that they are less toxic (Table 3). The chemotherapeutic effect of IA, IH and SMIH was studied in macrophages infected with promastigotes of *L*. *(L*.*) infantum chagasi* followed by treatment with the immucillins. When compared to untreated controls, a 74% reduction in the number of intracellular amastigotes was detected in macrophages treated with IA at 0h (Fig 7C). IA was also slightly superior to IH (70%), SMIH (70%) and to the gold standard drug Glucantime (70%) (Fig 7C). When macrophages were treated daily with the immucillins, the chemotherapeutic potential of IA increased to 95% (Fig 7D). In this protocol IA and other inhibitors were equally effective. The EC50 values for inhibition of replication of intracellular amastigotes were determined (Table 3). IA and IH showed the highest potency while SMIH and Glucantime exhibited similar EC50 values. These values determined higher selectivity indexes for IA and IH, which were followed by SMIH and Glucantime.

**Table 3:** Inhibitory concentrations of Immucillins and Glucantime against intracellular amastigotes of *L*. *(L*.*) infantum chagasi* and citotoxicity to macrophages.

Drugs	CC50 = μM (CI 95%)	EC50 = μM (CI 95%)	Selectivity index (CC50 value/ EC50 value)
**IA**	900 (400–650)	2.39 (1.58–5.58)	377
**IH**	950 (300–690)	3.69 (1.82–5.99)	257
**SMIH**	800 (400–500)	7.10 (7.03–7.17)	113
**Glucantime**	700 (400–510)	7.10 (7.01–7.19)	99

CC50 and EC50 were determined by linear interpolation after addition of the drugs at 0 h (10 μM, 500 μM and 5mM for IC50) and (0.5 μM, 5 μM and 10 μM for EC5

## Discussion

The nucleoside hydrolase from *Leishmania (L*.*) donovani*, cloned in the pET-22 expression vector, was described before by Cui et al. [29] as a non-specific inosine-uridine preferring NH. The NH36 is a non-specific adenosine, uridine, inosine and cytidine preferring nucleoside hydrolase, with a 3.6 and 1.4 fold increased catalytic rate for uridine (*k*_cat_ = 9.5 s^-1^) and inosine (*k*_cat_ = 7.6 s^-1^), respectively [29]. When compared to NH of *L*. *(L*.*) major* [13], also an inosine-uridine preferring NH, the NH36 of *L*. *(L*.*) donovani*, shows an 11 fold decreased catalytic turnover for inosine (*k*_cat_ = 119 s^-1^), similar levels for uridine (*k*_cat_ = 32 s^-1^) and 6 and 36 fold increased catalytic rates for adenosine (*k*_cat_ = 0.57) and cytidine (*k*_cat_ = 0.36), respectively [13]. This confirms that *Leishmania (L*.*) donovani* NH36 displays a wider range of activity on naturally occurring nucleosides.

We described the inhibitory effect of immucillins on the *in vitro* multiplication of promastigotes of *L*. *(L*.*) infantum chagasi and L*. *(L*.*) amazonensis* induced by micromolar concentrations of IA, IH, SerMe-ImmG [28] and DIG (DADMe-ImmG), or by a 10 fold lower concentration of the second generation acyclic immucillin DIH (DADMe-ImmH). SMIH (SerMe-ImmH), is a third generation acyclic immucillin [28] and was extremely inhibitory at a nanomolar concentration. These results suggest a potential future use of these agents in the treatment of VL and CL. We recently described a 93% of identity between the amino acid sequences of NH36 and the NH A34480 of *L*. *(L*.*) amazonensis* [37], which explains the similar inhibitory activity of immucillins on both nucleoside hydrolases. Although several nucleoside hydrolases have been predicted for the genome of *L*. *(L*.*) amazonensis* [38], to our knowledge, that of NH36 represents the single description of NHs in the *L*. *(L*.*) donovani/ L*. *(L*.*) infantum* complex [29–31, 39].

We demonstrated the inhibitory effect of immucillins on promastigote replication in Schneider’s supplemented insect medium, which contains serum in its composition. Inhibition of multiplication was obtained, in spite the presence of adenosine, a favored substrate of NH36. Other purines or pyrimidines are probably also present in this complex medium. Additionally, IA was able to inhibit promastigote replication in the RPMI medium which completely lacks nucleobases, nucleotides or nucleosides, and this inhibition was bypassed by the addition of adenosine, suggesting that immucillin IA might act on the parasite nucleoside hydrolase activity.

In the sand fly vector, promastigotes inhabit the insect gut and transmit the initial infection. In the host, amastigotes replicate inside the macrophages maintaining the infection, causing the disease and are the real target of chemotherapy. Therefore, the inhibition of replication of intracellular amastigotes is the most important target for chemotherapeutic purposes. Our results show strong chemotherapeutic effects of IA, IH and SMIH, that decrease the intracellular amastigote parasite load by 74–95% when used at low micromolar concentrations. Treatments of *L*. *(L*.*) infantum chagasi* promastigotes with IA, IH and SMIH induced alterations in vital parasite structures that suggest irreparable metabolic damage that could lead to the parasite death.

The ability of *Leishmania* to survive inside insect or mammalian hosts depends of its capability to sense and adapt to environmental changes [40]. *L*. *(L*.*) donovani* can survive in a purine deprived media for three months because it acquires purine-transporters and surface enzymes that undergo “up-regulation”, as a metabolic reprogramming during purine starvation [40]. Our results showed that, some of the immucillins were capable of inhibiting growth at 0–48 h, but growth inhibition was not complete at 72 to 96 h. Parasite could alter its purine metabolism or transport to reduce inhibitor efficacy by 96 h. This effect may explain why, repeated additions of immucillins in the daily protocol were needed to completely hall the parasite growth.

The inhibitory effect of immucillins in the context of leishmaniasis is impressive considering that Glucantime, the recommended standard drug, reached the IC50 for human strains of *L*. *(L*.*) infantum chagasi* at 1000 fold greater concentrations (0.43–5.76 mM) and showed even higher values against *Leishmania* strains isolated from dogs (19.97–32.27 mM) [41].

A crucial result of this investigation is the characterization of the inhibitory effect of immucillins on the amastigote replication inside the macrophages of a mammalian host. Amastigotes are the *Leishmania* parasite stage responsible for the development of leishmaniasis in humans, rodents and dogs and are therefore the important drug target. While a 74% inhibition of intracellular multiplication was achieved using a single IA 10 μM dose, the daily addition of IA, IH and SMIH was extremely effective, reducing by 95% the parasite load. Using a single dose, the treatment with IA showed the highest potency while no significant differences were found between IH, SMIH and Glucantime, which gave 70% growth inhibition. An important physiological finding is that either a single dose or the daily addition of three doses of each drug at 10 μM did not cause any decrease in macrophage viability but did give potential anti-parasite activity. Thus, the result indicates a new potential use of immucillins in therapy of leishmaniasis. *Leishmania* therapy. A recent study on the effect of Palladacycle complex on *L*. *(L*.*) amazonensis* replication showed that this drug destroyed axenic promastigotes and intracellular amastigotes *in vitro* at nanomolar concentrations while displaying 10-fold less toxicity than Glucantime to macrophages [42].

Our results gain impact if compared to other reports showing that much higher concentrations of the presently commercial available drugs, of known toxicity, are required in order to achieve the IC50 against intracellular amastigotes. Morais-Teixeira et al., [43] described that a 348 μM concentration of Glucantime was needed to achieve the IC50 against replication of amastigotes of *L*. *(L*.*) infantum chagasi* and 62.58 μM to inhibit amastigotes of *L*. *(L*.*) amazonensis*. Morais-Teixeira et al. [44] reported also higher concentrations Glucantime (1416 μM), Paramomycin (336.7 μM), and Azitromycin (19.9 μM) against intracellular amastigotes of *L*. *(L*.*) infantum chagasi*. The efficacies of immucillins are more compatible to the ones described for Miltefosine (4.4 μM) and Amphotericin (0.04 μM) also against amastigotes of *L*. *(L*.*) infantum chagasi* [44]. Similar IC50 values were also described for the treatment of *L*. *(L*.*) amazonensis* intracellular amastigotes [44]. Ramirez-Macias et al. [45], on the other hand, obtained the IC50 against intracellular amastigotes of *Leishmania infantum chagasi* at a 31.1 μM Glucantime concentration.

In the present study, IA showed a slight advantage in therapeutic efficacy above that of IH in inhibition of promastigote and amastigote replication. Although the immucillin IA was first described as an inhibitor of the nucleoside hydrolases of *L*. *(L*.*) major*, *Crithidia fasciculata and T*. *brucei brucei* [13], it was never tested before against *Leishmania* replication. Other immucillins assayed in this investigation were developed as inhibitory ligands of purine nucleoside phosphorylases [14, 28] and have not been reported for activity against NH36 nucleoside hydrolases nor against *Leishmania* parasites. Therefore, this study provides the first evidence of *in vitro* studies on the potential use of immucillins in leishmaniasis therapy.

IH (Forodesine) and DADMe-ImmH (Ulodesine) are potent inhibitors of human PNP that have been used in human clinical trials against leukemia and gout [28]. Low nucleoside levels in T cells of patients with Acute Lymphoblastic leukemia make them more sensitive to Forodesine inhibition than normal human lymphocytes [47]. IH [46] and DADMe-Immucillin-G [14] are powerful inhibitors of both human and malarial PNPs and cause purine starvation and death of *Plasmodium* parasites. Our study expands the potential use of this inhibitor family to the potential therapeutic efficacy and low toxicity against visceral leishmaniasis. Although additional *in vivo* studies are needed, to elucidate the impact of Forodesine on T and B cells of patients with visceral leishmaniasis, our results are encouraging considering that IH (Forodesine) is orally available, had already undergone safety trials in humans, and thereby shortens the time required until trials could begin for use against leishmaniasis.

Taken as a whole, these results are promising, considering the increased incidence of human VL cases caused by parasites resistant to Glucantime [10] and the strong toxicity determined by this therapy in the sensitive cases [6–8, 48, 49].

The development of new drugs to treat leishmaniasis is urgent, mainly because the currently existing drugs were developed for other indications, have invasive routes of administration, and have many adverse effects and therapeutic unresponsiveness. The immucillins should be the target of therapeutic studies, since it is reasonable to assume that effects seen at the cellular level might be recapitulated in humans.

## Supporting Information

S1 Fig NH36 purification by NiNTA column chromatography. Electrophoresis was performed in 12% polyacrylamide gels stained with Commassie blue. In (A) from left to right: BioRad molecular weight standards (10, 15, 20, 25, 37, 50 e 75 KDa) (Mw), 10 μL of bacterial supernatant loaded in the column (BS), 10 μL of the fraction eluted with elution buffer 50 (mM PO_4_K_2_, 300 mM Na Cl, pH: 8.0) (EF), 10 μL of the fractions obtained with elution buffer containing 50–300 mM imidazol, 10 μL of whole supernatant and 10 μL molecular weight standards (M). Elution of the purified NH36 is starts with 150 mM (*).
(TIF)

S2 Fig HPLC analysis. (A) An aliquot of 300 μl of Schneider´s insect medium supplemented with FBS was concentrated to dryness, resuspended in 0.1mL of a 50mM KH_2_PO4, 4mM tetra-n-butylammonium bromide (TBAB), 10% methanol solution, pH 6.0, and injected into a C-18 reverse-phase column (Rexcrom, 25 cm_4.6mm, Regis Technologies Inc., IL) coupled to an LC10AS-HPLC model (Shimadzu) through a 50-mL loop. Adenosine was separated using a 1ml/min flow rate (retention time: adenosine, 8.5 ± 0.1 and detected by UV spectroscopy at 254 nm) and its concentration was calculated by peak integration in comparison with the standard concentration of adenosine.
(TIF)
