# Supplementary figures and images for: Immucillins Impair Leishmania (L.) infantum chagasi and Leishmania (L.) amazonensis Multiplication In Vitro
Source: PLoS One. 2015 Apr 24;10(4):e0124183. doi: 10.1371/journal.pone.0124183 (PMC4409337; doi:10.1371/journal.pone.0124183)

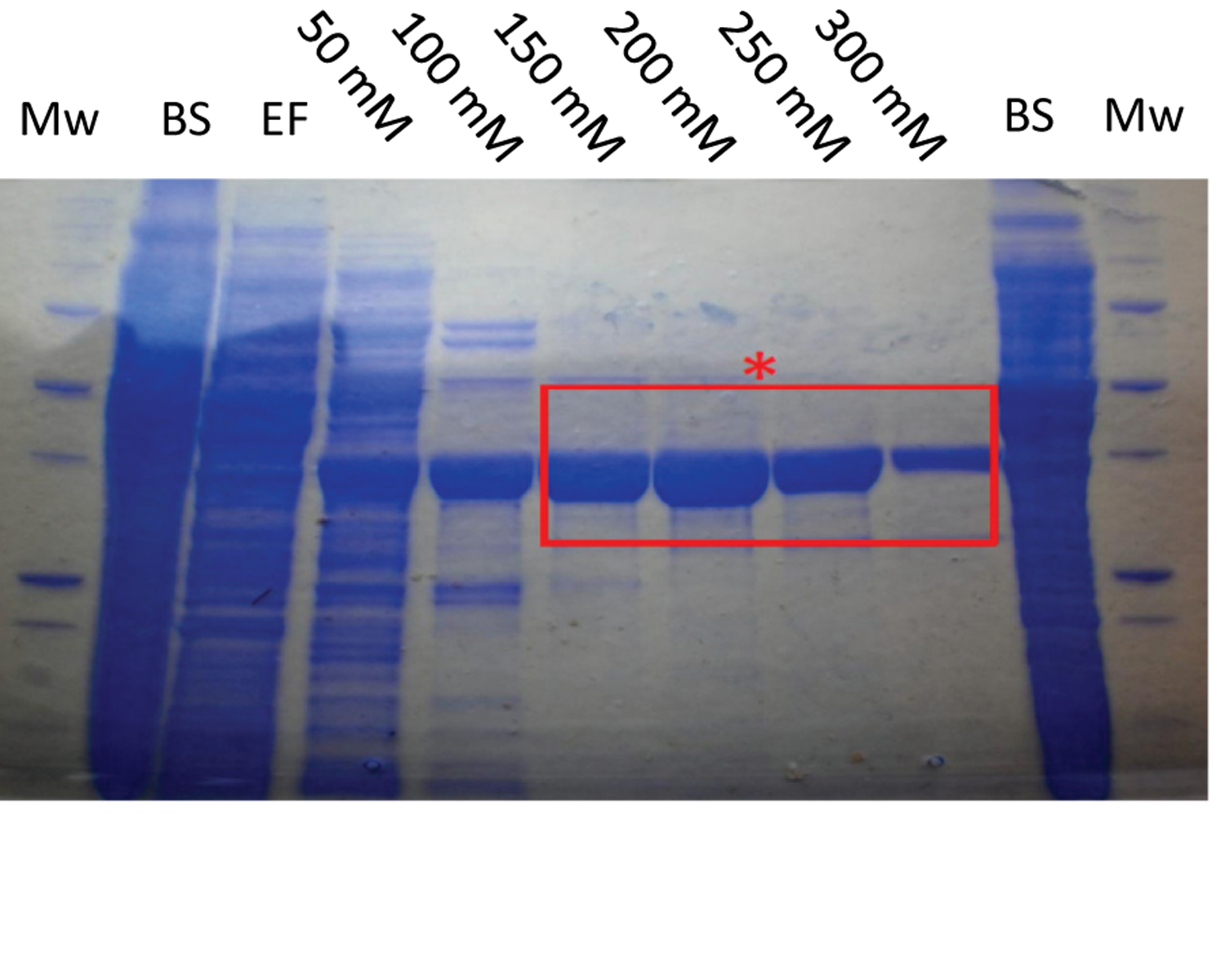

Supplement: S1 Fig — Electrophoresis was performed in 12% polyacrylamide gels stained with Commassie blue. In (A) from left to right: BioRad molecular weight standards (10, 15, 20, 25, 37, 50 e 75 KDa) (Mw), 10 μL of bacterial supernatant loaded in the column (BS), 10 μL of the fraction eluted with elution buffer 50 (mM PO4K2, 300 mM Na Cl, pH: 8.0) (EF), 10 μL of the fractions obtained with elution buffer containing 50–300 mM imidazol, 10 μL of whole supernatant and 10 μL molecular weight standards (M). Elution of the purified NH36 is starts with 150 mM (*). (TIF) [file pone.0124183.s001.tif]

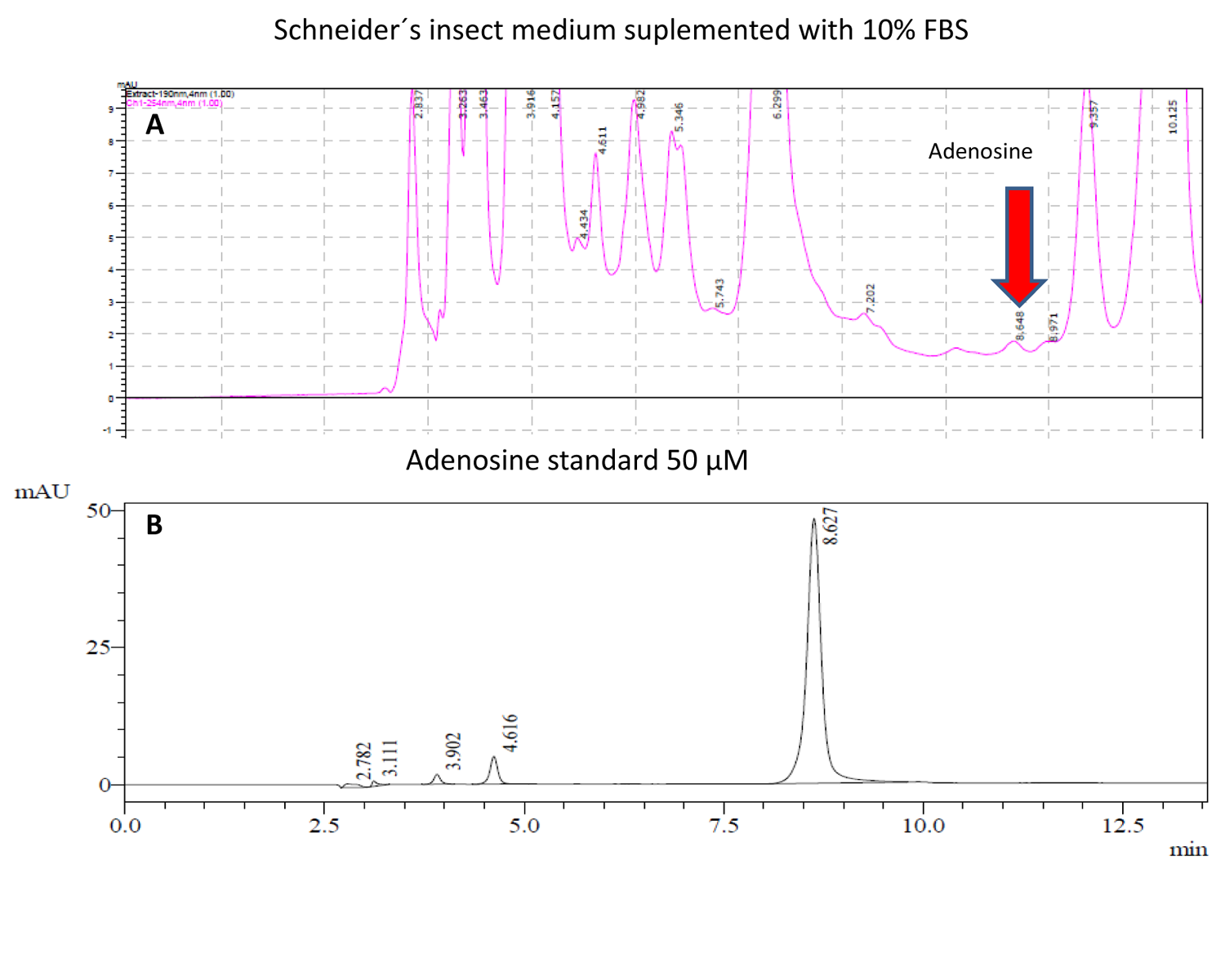

Supplement: S2 Fig — (A) An aliquot of 300 μl of Schneider´s insect medium supplemented with FBS was concentrated to dryness, resuspended in 0.1mL of a 50mM KH2PO4, 4mM tetra-n-butylammonium bromide (TBAB), 10% methanol solution, pH 6.0, and injected into a C-18 reverse-phase column (Rexcrom, 25 cm_4.6mm, Regis Technologies Inc., IL) coupled to an LC10AS-HPLC model (Shimadzu) through a 50-mL loop. Adenosine was separated using a 1ml/min flow rate (retention time: adenosine, 8.5 ± 0.1 and detected by UV spectroscopy at 254 nm) and its concentration was calculated by peak integration in comparison with the standard concentration of adenosine. (TIF) [file pone.0124183.s002.tif]
